# Neural networks involved in learning lexical-semantic and syntactic information in a second language

**DOI:** 10.3389/fpsyg.2014.01209

**Published:** 2014-10-30

**Authors:** Jutta L. Mueller, Shirley-Ann Rueschemeyer, Kentaro Ono, Motoaki Sugiura, Norihiro Sadato, Akinori Nakamura

**Affiliations:** ^1^Institute of Cognitive Science, University of OsnabrückOsnabrück, Germany; ^2^Department of Neuropsychology, Max Planck Institute for Human Cognitive and Brain SciencesLeipzig, Germany; ^3^Department of Psychology, University of YorkYork, UK; ^4^Department of Clinical and Experimental Neuroimaging, National Center for Geriatrics and GerontologyObu, Japan; ^5^Human Brain Research Center, Graduate School of Medicine, Kyoto UniversityJapan; ^6^Institute of Development, Aging and Cancer, Tohoku UniversitySendai, Japan; ^7^Department of Cerebral Research, National Institute for Physiological SciencesOkazaki, Japan

**Keywords:** word learning, syntactic learning, fMRI, plasticity, second language

## Abstract

The present study used functional magnetic resonance imaging (fMRI) to investigate the neural correlates of language acquisition in a realistic learning environment. Japanese native speakers were trained in a miniature version of German prior to fMRI scanning. During scanning they listened to (1) familiar sentences, (2) sentences including a novel sentence structure, and (3) sentences containing a novel word while visual context provided referential information. Learning-related decreases of brain activation over time were found in a mainly left-hemispheric network comprising classical frontal and temporal language areas as well as parietal and subcortical regions and were largely overlapping for novel words and the novel sentence structure in initial stages of learning. Differences occurred at later stages of learning during which content-specific activation patterns in prefrontal, parietal and temporal cortices emerged. The results are taken as evidence for a domain-general network supporting the initial stages of language learning which dynamically adapts as learners become proficient.

## Introduction

Learning a new language requires the mastery of many skills, including the ability to recognize and use novel words and the utilization of novel syntactic structure. Clearly the ability to recognize the meaning of words and the ability to extract meaning from syntactic structure are very different cognitive processes, yet both are critical to becoming a proficient user of a language. In addition, language learners in natural linguistic contexts are confronted with novel words and novel syntactic structures simultaneously, and must learn to extract the relevant information for both domains from the same signal. While much research on the neural basis of language learning has focused on the acquisition of either novel words or syntactic structures, few studies have attempted to explain how brain mechanisms supporting the two domains might compare. The current study attempts to address this gap by investigating what brain areas are involved in the simultaneous learning of words and syntactic structures in a new language.

A growing number of neurophysiological studies have investigated the individual components of language learning (e.g., recognizing and producing language-specific phonotactic, semantic, or syntactic information) in isolation. For example, learning of syntactic rules has been assessed in artificial grammar learning (AGL) paradigms in which no semantic or contextual information is provided to the learners (Tettamanti et al., 2002; Musso et al., 2003; Opitz and Friederici, 2003, 2004). While some of these studies used an artificial grammar that contained language-like phrase structure rules (Opitz and Friederici, 2003, 2004), others used real existing languages as the learning basis (Tettamanti et al., 2002; Musso et al., 2003). The most important finding reported consistently in all of these studies is that the left inferior frontal gyrus (IFG) and surround prefrontal areas, i.e., Broca's area, showed increasing activation as learning proceeded. Intriguingly, this was the case only for syntactic rules that are relevant for human languages and not for non-linguistic rules (Tettamanti et al., 2002; Musso et al., 2003). The activation for learning of syntactic rules in a natural language was located in BA 45 (Musso et al., 2003), and in purely artificial grammars it was located in a more posterior portion of the IFG in BA44/6 (Opitz and Friederici, 2003, 2004). In accordance with these findings two longitudinal studies on second language (L2) sentence comprehension found increasing activation of posterior portions of the IFG from earlier to later stages of language training (Indefrey, 2006; Newman-Norlund et al., 2006). Thus, AGL as well as L2 learning appear to result in increasing activity within the left IFG. Thus, it appears that the left inferior frontal cortex comes into play as knowledge about the underlying regular structure becomes available.

Experiments investigating the acquisition of novel words have used a number of different experimental protocols, both at the single word and at the sentence level. Imaging studies which tested the acquisition of novel phonological forms by repeated presentations of pseudowords report the involvement of left IFG and precentral gyrus, the superior temporal gyrus, the (pre-) supplementary motor area (SMA) and the cerebellum to be involved during phonological acquisition (Rauschecker et al., 2008; Paulesu et al., 2009). A study focusing on consolidation effects during the learning of words reported initial involvement of the hippocampus and modulations of the superior temporal cortex after consolidation (Davis et al., 2009). Other studies incorporated semantic meaning in their pseudoword-learning task, thereby enabling the acquisition of lexical and semantic information (Breitenstein et al., 2005; Mestres-Missé et al., 2008, 2009). Breitenstein et al. (2005) applied an audio-visual association paradigm in which participants were exposed to concurrent presentations of pictures and words. Repeated presentations of picture-word pairings led to decreasing activation in the left hippocampus and the left fusiform gyrus and to increasing activation in the left parietal lobe. In contrast, the studies of Mestres-Missé et al. used a paradigm in which triplets of sentences were presented during which the meaning of a novel word became increasingly clear through both contextual semantic and syntactic information. The network found to be related to meaning acquisition under this learning condition comprised the left inferior and middle frontal gyri, the middle and superior temporal gyri, the pre-SMA, bilateral caudate nuclei, the left thalamus and the left parahippocampal gyrus (Mestres-Missé et al., 2008, 2009).

From the neurophysiological evidence available it is unclear whether the learning of words and sentence structure in the adult brain are based on the same or different brain mechanisms. Specifically, the prefrontal cortex seems to react differently depending on the information in focus: with decreasing or stable activation over time when phonological aspects of words were crucial (Mestres-Missé et al., 2008, 2009; Rauschecker et al., 2008; Paulesu et al., 2009) and increasing activation over time when syntactic information was crucial (Tettamanti et al., 2002; Musso et al., 2003; Opitz and Friederici, 2003, 2004). Further, the learning of lexical-semantic aspects of words additionally seem to involve more widespread areas including subcortical, temporal and parietal structures (Breitenstein et al., 2005; Mestres-Missé et al., 2008, 2009; Rauschecker et al., 2008; Paulesu et al., 2009). From the above mentioned studies it is not clear whether the learning of words vs. syntax generally rely on identical or different brain mechanisms and whether seemingly different patterns of activation are due to the use of different learning paradigms. It seems likely, that the activation patterns even converge if identical cues for learning are available. This hypothesis is inspired by experiments on associative learning which have shown that domain-general mechanisms of control, such as working memory and selective attention, guide initial stages of learning regardless of the linguistic or non-linguistic nature of the material (cf. Chein and Schneider, 2005, for review). More specific to the domain of language, Zhang and Wang (2007) have proposed that initial stages of speech learning are guided by general attentional resources and move toward more specialized, differentiated activation patterns as learners become proficient. Alternatively, it is possible, that some regions specifically support word-learning or syntax learning from the start. We will refer to these two possibilities by the terms *learning generality hypothesis* and *learning specificity hypothesis*.

Here, we investigate the neural correlates of both the learning of words and the learning of sentence structure in adults by using an audio-visual sentence-picture matching task in which extralinguistic visual context is used as an unambiguous cue to the interpretation of a spoken sentence. Participants were trained in the scanner to recognize and use a number of novel words and a novel syntactic structure in a language they were previously unfamiliar with (German). Functional MRI was used to assess what brain areas were activated during the initial presentation of novel stimuli (i.e., at the beginning of the experiment) and how these activation patterns changed over time (i.e., at the end of the experiment). Importantly we investigated the location of both domain-general learning effects (i.e., areas sensitive to learning over time irrespective of the condition) and domain-specific effect (i.e., areas that were primarily involved in the learning of either lexical-semantic or syntactic information).

## Materials and methods

### Participants

Twenty right-handed native Japanese (10 females), aged between 20 and 26 years (mean: 22.3 years), with no previous experience of the German language participated in the experiment. The study was approved by the ethical committees of both the National Center for Geriatrics and Gerontology, Obu, and the National Institute for Physiological Sciences, Okazaki, and written informed consent was obtained from all of the participants prior to the experiment.

### Stimuli

#### Sentence stimuli

The stimuli were spoken sentences taken from a miniature version of German which comprised 27 words. The words were four nouns referring to professions (*Maler, Schüler, Priester, Zahnarzt*; “painter, pupil, priest, dentist”), 14 nouns referring to objects (*Teller, Pilz, Strauß, Besen, Käse, Reifen, Würfel, Spiegel, Stiefel, Korb, Topf, Schal, Kamm, Schirm*; “plate, mushroom, bouquet, broom, cheese, tire, dice, mirror, boot, basket, pot, scarf, comb, umbrella”), three determiners (*der, dem, den*; nominative definite determiner, dative definite determiner, accusative definite determiner), two auxiliaries (*hat, wurde*; “was, has”), one temporal adverb (*gestern*; “yesterday”), one preposition (*vom*, “by”), and two verbs (*gegeben, gezeigt*; “given, shown”). All of the sentence stimuli for both the pre-scanner training and the within-scanner training were created from this subset of words. All sentences contained reference to two people (e.g., painter and priest) performing one of two actions (giving or showing) on one of 14 objects (plate, mushroom, etc.). Sentences could be in either the active (see sentence 1) or the passive (see sentence 2) voice. Passives were chosen because they express the same meaning as the corresponding active sentences and could thus, be learned from identical pictures. The basic principle of forming passive constructions are comparable across Japanese and German: the different syntactic roles are indicated by case marking and the verb form changes. The main differences across the languages are, that in German, passives are built by inserting the inflected form of the auxiliary “werden” (in our case the past tense form “wurde”) together with the participle form of the main verb while in Japanese only the suffix of the verb is changed.

1. *Gestern hat der Schüler dem Priester den Teller gezeigt*.
  Yesterday the pupil showed the plate to the priest.
2. *Gestern **wurde der Teller vom Schüler dem Priester** gezeigt*.
  Yesterday the plate was shown to the priest by the pupil.

Our items contain speech sounds that are difficult to identify correctly for Japanese native speakers, e.g., consonant clusters and r and l sounds (cf. Dupoux et al., 1999). This was the case across the whole stimulus material and not specific to any of the conditions. Thus, learning about the non-native sound system was an integral part of our learning task.

#### Pictures

**Figure 1:**
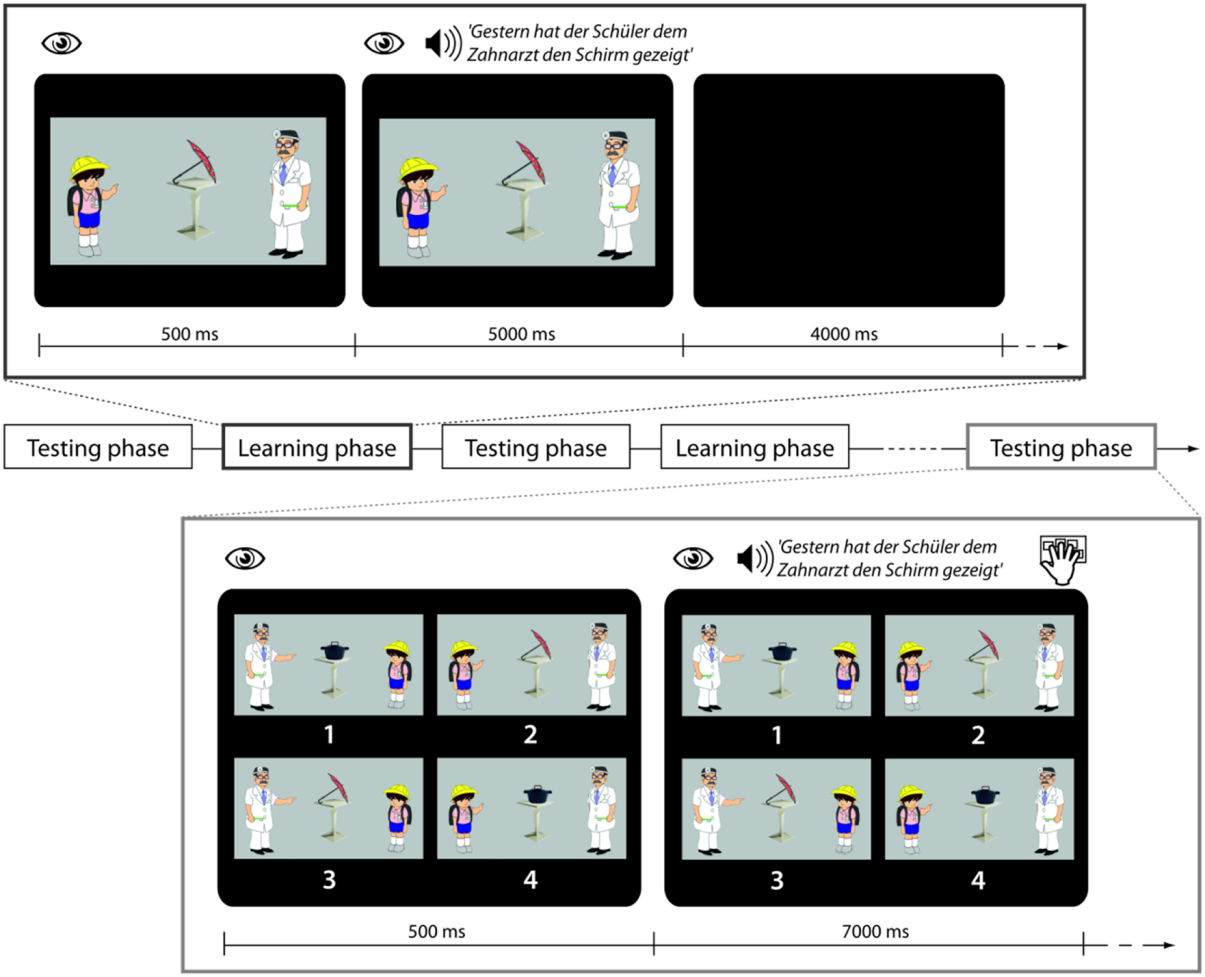
**Trial structure in learning and testing phases**. During a trial in the learning phase participants are looking at a picture and then auditorily presented a sentence with the task to use the picture for comprehension. During a trial in the testing phase participants are looking at four pictures and then presented with a sentence which they are asked to match with the right picture.

Colored drawings that depicted each of the possible combinations of actions described verbally were created (see Figure 1). Pictures of each individual agent/object could be presented in isolation or embedded in an action sequence. In addition a number of scrambled drawings, i.e., drawings in which agents and objects were unrecognizable were also created.

### Procedure

Participants underwent two extensive training sessions, one before scanning and one during scanning. The tasks were programmed and presented using Presentation (Neurobehavioral Systems, Inc.). The stimulus delivery during the pre-scanning training was done on notebooks with headphones and during the scanning session with headphones and a mirror above the participants' heads showing the image of a computer screen outside the scanner.

#### Pre-scanner session

One day before fMRI scanning participants were trained on a basic version of miniature German comprising a subset of the total stimuli. Specifically, the four agent nouns, four of the 14 objects, the three determiners, one of the possible auxiliaries, the temporal adverb and one of the two verbs were taught to participants in three training stages. This subset of the total stimuli could be combined into 96 possible sentences using one syntactic structure (i.e., active voice). During the first stage object names were presented auditorily along with their corresponding pictures. During the second stage the grammatical properties of active sentences including case marking in German were explained explicitly (e.g., by presenting a single case marked noun phrase together with a picture where the corresponding part, e.g., the actor, was highlighted). During the third stage whole sentences were presented along with four pictures of possible events, and participants had to decide which of four presented pictures corresponded to the sentence (four-alternative forced choice task). During the last stage participants performed the four-alternative forced choice task under time constraints. Direct feedback about the accuracy of the answer was given after every judgment during both sentence judgment tasks. When they reached the criterion of 95% correct answers in the speeded sentence picture matching task, they were scheduled for the fMRI experiment on the following day.

#### Within scanner session

During fMRI scanning participants were exposed to a sequence of five testing blocks (TB1–TB5) and four learning blocks (LB1–LB4) which were presented in alternation (see Figure 1 for schematic presentation and example stimuli).

#### Within scanner training blocks

Each learning phase contained 10 sentences belonging to one of four conditions, i.e., 40 sentences in total. In the Familiar Condition (F) sentences containing words that had been trained the previous day and using the familiar active sentence structure with which participants had been made familiar were presented. In the Novel Word Condition (W) sentences contained reference to a novel object that had not been trained the previous day. (The 10 novel objects were *Käse, Reifen, Würfel, Spiegel, Stiefel, Korb, Topf, Schal, Kamm, Schirm*, cheese, tire, dice, mirror, boot, basket, pot, scarf, comb, umbrella'). All W sentences used syntactic structures that had already been learnt. In the Novel Syntactic Structure Condition (S) sentences in the passive voice were presented. This involved the introduction of the novel auxiliary *wurde*, “was” and the preposition *vom*, “by.” Lastly in the Perceptual Control Condition (R) the familiar sentences were played backwards. In conjunction with each sentence (F, W, S) a picture was presented showing the relevant agent involved in an action event with the relevant object. Durations of the sentences across conditions were very similar (F condition: 4.64 s, SD 0.24 s; W condition: 4.60 s, SD 0.24; S condition: 4.78 s, SD 0.24 s). All sentences were normalized to the same mean intensity. The sound level during presentation was adjusted individually to a comfortable level in the presence of scanner noise. Participants were explicitly instructed to use the picture to figure out the meaning of the spoken sentences. In conjunction with the R sentence stimuli a scrambled picture was presented. All S sentences contained familiar words, i.e., there were no sentences in which both a novel word and a novel structure were introduced at the same time.

#### Within scanner testing blocks

During testing phases participants listened to 30 sentences belonging to the F, W, and S Conditions in pseudorandomized order and to perform a sentence-picture matching task as in the pre-scanning training. In order to prevent strategic gaze movements, the pictures were organized in randomized positions.

### fMRI data acquisition

Imaging was performed on a 3T scanner (Siemens Allegra). A high-resolution anatomical T1-weighted image was acquired by magnetization-prepared rapid gradient-echo (MPRAGE) imaging (*TR* = 2.5 s; *TE* = 4.38 ms; *FA* = 8; 256 × 256 matrix; 192 slices; voxel dimensions = 0.75 × 0.75 × 1 mm) for each participant. Functional MRI scanning was carried out using a T2^*^-weighted BOLD sensitive gradient-echo echo-planar imaging sequence (*TR* = 2 s, *TE* = 30 ms, *FOV* = 19.2 cm, 64 × 64 matrix, resulting in an in-plane resolution of 3 × 3 mm). Twenty slices (thickness: 4 mm with an interslice gap of 1 mm) covering the whole brain were acquired. Anatomical and functional images were positioned parallel to AC-PC. Five functional runs were collected, with the first run containing the data from the first testing block and each of the subsequent runs containing the data from the following learning and testing block. Between the runs participants could take a short rest. The whole experiment had a duration of about 60 min.

### fMRI analysis

Data processing was performed using SPM8 (available at http://www.fil.ion.ucl.ac.uk/spm/). Preprocessing of the time series involved: motion correction (rigid-body realignment), a slice-time correction using sink interpolation, a spatial smoothing (Gaussian kernel with 7 mm FWHM), and, baseline correction using a temporal high-pass filter (cutoff frequency: 1/120 Hz). The time series were co-registered with high-resolution T1 images that were acquired before the functional measurement. To achieve an optimal match between the T1 image and the functional time series, co-registration was performed separately in each of the five functional runs. Functional images were then normalized to MNI space using linear and non-linear normalization.

The statistical evaluation used a mass-univariate approach based on the General Linear Model as implemented in SPM8. The design matrix was generated with a box-car function, convolved with the hemodynamic response function. Serial correlations in the data were dealt with by applying an autoregressive model (AR1) during parameter estimation. On the first level, individual contrast-images, i.e., estimates of the raw-score differences between each learning condition (W, S) and the familiar condition (F) were calculated separately for each learning block [e.g., (W LB1—F LB1) and (S LB1—F LB1)]. These contrasts show the processing of novel vs. familiar sentences at specific stages of learning. The single-participant contrast-images were then entered into a second-level random effects analysis. The group analysis consisted of a 2 × 4 ANOVA including the factors CONDITION (novel word vs. novel sentence structure) and BLOCK (learning block 1 through learning block 4) across the contrast images for all participants. The combination of voxel-based thresholds with a minimum cluster-size has been argued to improve the statistical power (Forman et al., 1995). We applied this double-threshold approach to protect against false positive activations, considering an area to be activated only if it comprised a volume greater than or equal to 648 mm^3^ (24 voxels) and had a Z-score of greater than 3.09 (*p* < 0.001, uncorrected). This non-arbitrary voxel cluster size was determined by using the program AlphaSim implemented in the AFNI software (Cox, 1996), and corresponds to a cluster corrected threshold of *p* < 0.05. Significant areas that appeared in the ANOVA were used as a mask for pairwise comparisons (*t*-tests) of different factor levels that were conducted to specify simple main effects. Figures show the resulting thresholded activation maps overlaid onto the standard MNI brain included in SPM8.

## Results

### Behavioral results

**Figure 2:**
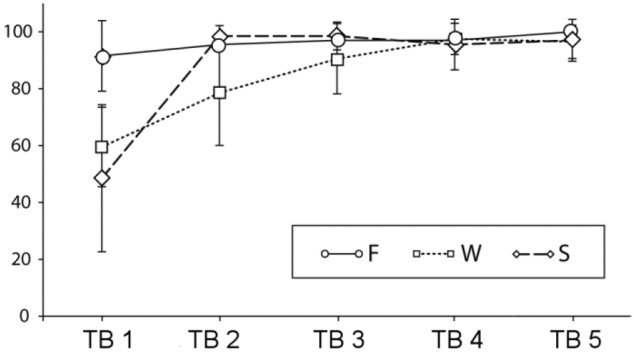
**Percent correct answers and standard deviations in the sentence-picture matching task across testing blocks (TBs) (F, familiar condition; W, novel word condition; S, novel sentence structure condition)**.

Figure 2 shows the results of the sentence-picture matching task during testing blocks (TB1 to TB5). Mean accuracy rates in the F condition were constantly high [TB1–TB5: 91.1 (SD 12.3), 95.5 (SD 6.0), 97 (SD 5.7), 97 (SD 7.3), 100(SD 0)], in the W condition a gradual increase could be seen [TB1–LB5: 59.5 (SD 13.9), 78.5 (SD 18.4), 90.5 (SD 12.3), 97.5 (SD 5.5), 96.5 (SD 5.9)], and in the S condition an increase from the first to the subsequent blocks [TB1–LB5: 48.5 (SD 25.8), 98.5 (SD 3.7), 98.5 (SD 4.9), 95.5 (SD 8.9), 97 (SD 7.3)]. Behavioral data were assessed with an overall ANOVA and further step-down ANOVAs and *t*-tests. When more than four *t*-tests were conducted the critical alpha-level was adjusted according to the modified Bonferroni test suggested by Keppel (1991). This was only necessary for the F condition for which all possible 10 *post-hoc* comparisons were calculated.

The overall ANOVA revealed significant main effects of learning block [*F*_(4,76)_ = 91.61, *p* < 0.0001], of condition [*F*_(4,76)_ = 34.81, *p* < 0.0001] and a block by condition interaction [*F*_(8,152)_ = 22.72, *p* < 0.0001]. To follow up the interaction, we conducted separate ANOVAs for each condition. There was a significant increase in performance in the F condition [*F*_(4,76)_ = 4.29, *p* = 0.01]. *Post-hoc* tests revealed that the only reliable differences were between first and fifth testing block [*t*_(19)_ = 3.1, *p* = 0.005] and between second and fifth testing block [*t*_(19)_ = 3.3, *p* = 0.004]. In the W condition was a main effect of learning block [*F*_(4,76)_ = 42.83, *p* < 0.0001]. Four *t*-tests comparing all subsequent blocks with each other revealed reliable differences between the first and the second [*t*_(19)_ = 4.1, *p* < 0.001] the second and the third [*t*_(19)_ = 3.2, *p* < 0.01] and the third and the fourth testing block [*t*_(19)_ = 2.8, *p* = 0.01] while there was no significant difference between the last two testing blocks. For the S condition there was also a main effect of learning block [*F*_(4,76)_ = 64.04, *p* < 0.0001], however, when comparing the subsequent blocks to each other, only the difference between the first and the second testing block were significant [*t*_(19)_ = 8.7, *p* < 0.0001]. The *p*-values for the ANOVAS are Greenhouse-Geisser corrected. In sum, the results show a different speed of learning for the novel word and the novel sentence structure condition and a residual, slow learning process also for the familiar condition.

### fMRI results

The 2 × 4 factorial ANOVA including the factors CONDITION and BLOCK resulted in main effects CONDITION and BLOCK as well as in interactions between the two. As we are specifically interested in learning-related changes, we will report all effects including the factor BLOCK.

#### Main effect of learning block: learning-related activations across both learning conditions

**Figure 3:**
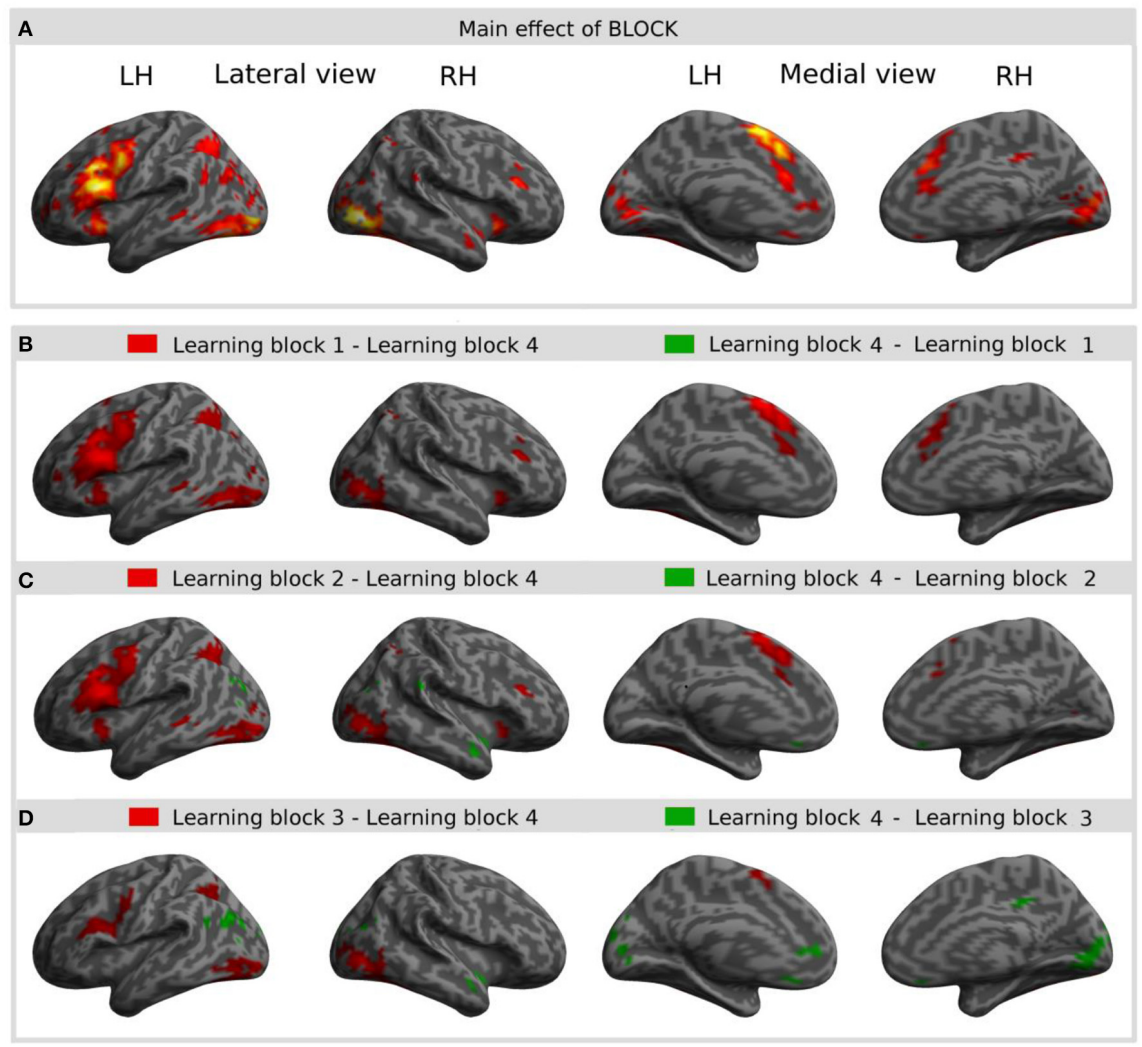
**Result of the main effect of the 2 × 4 ANOVA with the factors CONDITION and BLOCK and *post-hoc t*-tests to test simple main effects rendered on an inflated standard MNI cortical surface**. **(A)** Shows the main effect of the factor BLOCK (learning block 1–4). **(B–D)** show t-contrasts between the first **(B)**, the second **(C)**, and the third **(D)** learning block with the last learning block in order to illustrate the direction of changes over time. Colored areas represent the extent of activations, not statistical values of individual voxels.

**Figure 4:**
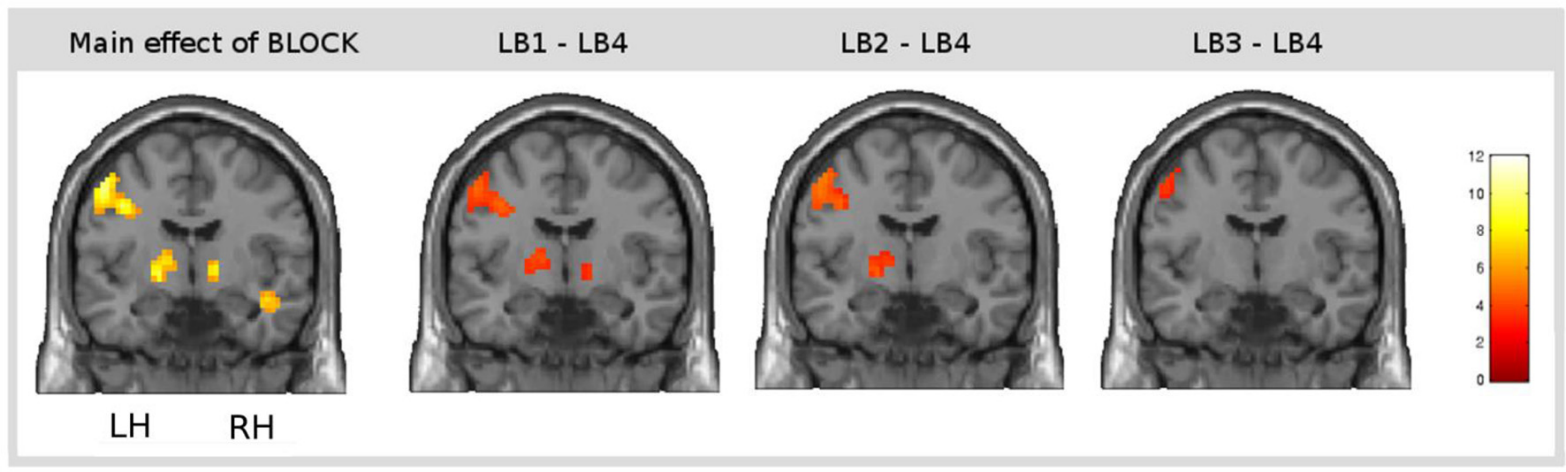
**Result of the main effect of the 2 × 4 ANOVA with the factors CONDITION and BLOCK and *post-hoc t*-tests to test simple main effects rendered on a coronal slice**. Bilateral basal ganglia showed learning-related changes over time. The main effect of the factor BLOCK (learning block 1–4) at the position *y* = −7, and, from left to right, contrasts between the first (LB1), the second (LB2), and the third learning block (LB3) with the last learning block (LB4) illustrate the direction of changes over time. The colorbar represents *z*-values.

The main effect of learning block revealed a widespread network of brain areas with activation changes across the four learning blocks (cf. Figures 3A, 4, Table 1). Pairwise contrasts of each learning block with the last learning block revealed that most changes were decreasing activations over time (cf. Figures 3B–D, 4, Table 1). The strongest decreasing activations were found in bilateral temporo-occipital and cerebellar areas and in left frontal and subcortical areas. In the right hemisphere frontal activation was observed too, but much less widespread. Additionally there was an involvement of left superior temporal sulcus, bilateral pre-SMA, cingulate cortex and superior parietal cortex. There were only a few areas that showed increasing activation over time. This was the case for bilateral middle temporal gyrus, right supramarginal gyrus and right anterior temporal lobe and medial frontal areas in the cuneus and orbitofrontal cortex. Subcortically, the pallidum was involved bilaterally (cf. Figure 4).

**Table 1:** **Brain regions, coordinates (MNI), and *z*-values of local maxima found for the main effect of BLOCK (learning block 1–4)**.

**Regions**	**±**	**BA**	**Zmax**	**voxel**	***x***	***y***	***z***
**LEFT HEMISPHERE**							
Frontal and subcortical areas	−	46/9	7.40	1687	−48	20	22
*Inferior frontal gyrus*	−	44			−54	14	7
*Inferior frontal gyrus*	−	45/47			−51	26	4
*Insula*	−	13			−36	17	−5
*Globus pallidus*	−				−18	−1	4
*Thalamus*	−				−12	−10	7
Frontopolar cortex	−	10	4.44	115	−24	50	−2
Pre-SMA	−	6	6.76	693	−3	11	58
*Cingulate gyrus*	−	32			9	26	31
Sup. temporal sulcus	−	22	4.00	38	−51	−37	1
Mid. temporal gyrus	+	39	4.65	490	−45	−79	25
Sup. parietal gyrus	−				−33	−55	49
**RIGHT HEMISPHERE**							
Insula	−	13	5.42	108	33	20	−5
Mid. frontal gyurs	−	9	4.88	80	42	29	28
Globus pallidus	−		4.21	80	15	−4	−2
Orbitofrontal cortex	−	11	3.91	51	0	38	−20
Sup. parietal gyrus	−	7	3.95	59	33	−58	49
Supramarginal gyrus	+	40	3.75	30	66	−43	25
Mid. temporal gyrus	+	21	3.74	44	42	−4	−20
Mid. temporal gyrus	+	39	3.90	34	54	−67	25
Post. cingulate gyrus	−	31	4.09	43	3	−34	43
Occipital and cerebellar areas	−	19	7.23	2532	45	−73	−5
*Cerebellum*	−				36	−64	−29
*Cerebellum*	−				−39	−70	−23
*Mid. temporal gyrus*	−	22			−51	−49	−8
*Occipital gyrus*	−	19			−39	−76	−14
*Cuneus*	−	17			3	−85	4

For large clusters across different regions additional local maxima are listed. Areas that show increasing activation over time (cf. Figure 3) are marked with +, areas that show decreasing activation over time with −.

#### Interaction of learning block and condition: specific activation for the learning of novel words

**Figure 5:**
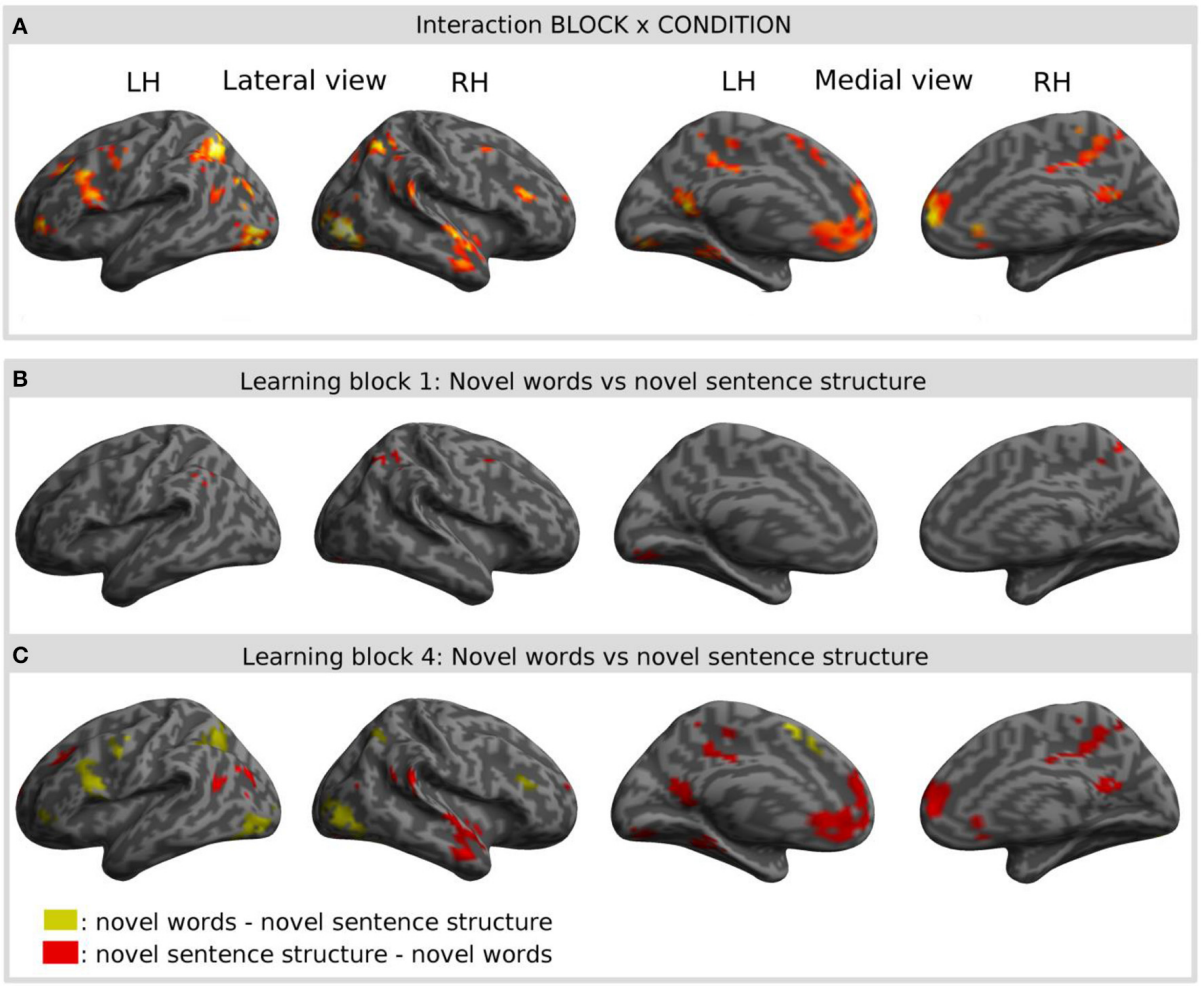
**Result of the interaction effect of the 2 × 4 ANOVA with the factors CONDITION and BLOCK and *post-hoc t*-tests to test simple main effects rendered on an inflated standard MNI cortical surface**. **(A)** Shows the interaction of the factor BLOCK (learning block 1–4) and CONDITION (novel word vs. novel sentence structure). **(B,C)** Show contrasts between the novel word and the novel sentence structure condition for the first **(B)** and for the last **(C)** learning block. Colored areas represent the extent of activations, not statistical values of individual voxels.

Interaction effects between the factors BLOCK and CONDITION were found bilaterally in frontal, parietal and temporal areas. The interaction effect is shown in Figure 5A and Table 2 and further contrasts (*t*-tests) between the W and the S condition within the first and the last learning block are shown in Figures 5B,C and Table 3. In the first learning block (Figure 5B) there was more activation of S condition compared to the W condition mainly in parietal and occipital areas. All further blocks, exemplified for the last learning block (Figure 5C), were characterized by more activation of the W condition compared to the S condition in a left fronto-parietal network and a right temporo-parietal network. In the last learning block there was also an increase for the S condition in medial frontal, posterior cingulate and temporal areas.

**Table 2:** **Brain regions, coordinates (MNI), and *z*-values of local maxima found for the interaction effect of BLOCK (learning block 1–4) × CONDITION (novel words vs. novel sentence structure)**.

	**BA**	**Zmax**	**voxel**	***x***	***y***	***z***
**LEFT HEMISPHERE**						
Left frontopolar cortex	10	4.64	68	−39	50	1
Inferior/Middle frontal gyrus	44/9	4.65	284	−48	17	28
Middle frontal gyrus	9	4.81	65	−24	26	31
Pre-SMA/cingulate	6/8	3.58	45	−3	20	46
Superior parietal gyrus	7	5.4	341	−36	−64	46
Middle temporal gyrus	39	4.86	75	−48	−76	25
Middle temporal gyrus	39	3.9	31	−42	−58	19
Parahippocampal gyrus	36	4.16	63	−27	−43	−14
Posterior cingulate	30	4.77	190	−6	−55	7
Inferior occipital gyrus	18	5.38	207	−33	−85	−5
Cuneus	18	4.52	113	−9	−79	−14
**RIGHT HEMISPHERE**						
Medial frontal gyrus	10	5.14	562	6	56	4
Middle frontal gyrus	46/9	4.61	56	48	32	25
Middle frontal gyrus	6	4.06	37	36	8	55
Superior parietal gyrus	7	5.06	224	36	−55	52
Middle temporal gyrus	21	5.31	282	54	−7	−17
Supramarginal gyrus	40	5.21	126	66	−46	22
Inferior occipital gyrus	19	5.75	366	45	−76	−2
Precuneus	7	4.82	313	3	−34	46
Cerebellum		4.15	37	9	−76	−26

**Table 3:** **Brain regions, coordinates (MNI), and *z*-values of local maxima found for novel words vs. novel sentence structure in each learning block within those regions that showed a BLOCK (LB1–LB4) × CONDITION (novel words vs. novel sentence structure) interaction effect**.

	**H**	**BA**	**Zmax**	**voxel**	***x***	***y***	***z***
**LB 1: NOVEL SENTENCE STRUCTURE > NOVEL WORDS**							
Sup. parietal gyrus	L	7	3.76	31	−45	−52	37
Cuneus	L	18	4.56	96	−15	−82	−17
Mid. frontal gyrus	R	6	3.64	25	33	5	58
Inf. parietal gyrus	R	40	3.72	86	36	−46	46
Precuneus	R	7	4.55	27	3	−61	55
**LB 4: NOVEL SENTENCE STRUCTURE > NOVEL WORDS**							
Mid. frontal gyrus	L	8	6.50	65	−24	26	42
Mid. temporal gyrus	L	39	>8	75	−48	−76	25
Mid. temporal gyrus	L	39	7.26	31	−42	−58	19
Lingual gyrus	L	18	5.99	75	−12	−82	−11
Parahippocampal gyrus	L	37	5.98	63	−27	−43	−14
Post. cingulate	L	23	7.24	190	−9	−58	13
Med. frontal gyrus	R	10	7.84	562	6	56	4
Mid. temporal gyrus	R	21	7.25	282	54	−7	−17
Precuneus	R	7	6.82	313	3	−34	46
Supramarginal gyrus	R	40	7.50	126	63	−46	22
**LB 2: NOVEL WORDS > NOVEL SENTENCE STRUCTURE**							
Frontopolar cortex	L	10	5.15	68	−36	47	−2
Inf./mid. frontal gyrus	L	44/ 9	6.29	258	−45	20	22
Pre-SMA	L	6	7.19	45	−6	23	40
Sup. parietal gyrus	L	7	4.15	154	−36	−52	46
Cerebellum	L		4.14	43	−33	−70	−26
Inf. occipital gyrus	R	19	4.92	96	33	−85	−2
Cerebellum	R		3.98	29	9	−79	−26
Cerebellum	R		5.15	126	36	−64	−29
**LB 3: NOVEL WORDS > NOVEL SENTENCE STRUCTURE**							
Inf./mid. frontal gyrus	L	44/9	4.34	133	−48	20	22
Pre-SMA	L	6	4.77	39	−3	8	58
Cerebellum	R		3.57	71	42	−73	−29
**LB 4: NOVEL WORDS > NOVEL SENTENCE STRUCTURE**							
Frontopolar cortex	L	10	4.18	33	−42	44	1
Inf./mid. frontal gyrus	L	44/9/6	6.44	241	−51	−1	46
Pre-SMA	L	6	6.89	43	−6	8	58
Sup. parietal gyrus	L	7	5.91	237	−33	−61	46
Inferior occipital gyrus	L	18	7.04	164	−33	−85	−5
Mid. frontal gyrus	R	46/9	4.44	43	48	32	28
Sup. parietal gyrus	R	7	4.27	91	36	−55	52
Inf. occipital gyrus	R	18	7.84	365	33	−85	−2

## Discussion

The present study investigated the short-term functional plasticity in the brain related to the learning of novel words and a novel syntactic structure from auditory linguistic input accompanied by extralinguistic context information. To our knowledge, this is the first time that the learning of both novel words and a novel sentence structure were investigated in a single experimental paradigm. Our results point to an overlapping brain network for initial steps of learning of both types of linguistic material, however, with emerging differences over time corresponding to the behavioral effects. While learning a novel sentence structure occurred immediately and fully in the first block, effects for the learning of words were spread over all four blocks. There was also a subtle learning effect in the familiar sentence condition, which served as a control for unspecific habituation effects in all comparisons.

The areas that were found for both the learning or words as well as sentence structure comprised a largely left lateralized network including inferior, middle and medial frontal cortices, temporal, parietal and subcortical areas. In the first learning block, for which behavioral learning effects were present for both learning conditions, there was almost no difference between learning of novel words and learning of novel syntactic structures. In the subsequent learning blocks, however, learning of novel words engaged prefrontal and parietal areas, and sentence structure learning recruited medial prefrontal areas, posterior cingulate, precuneus as well as temporal areas to a higher degree, although the performance levels were identical across both learning conditions. In the following we will discuss the common areas in the initial stages of learning and the emerging differences between word and sentence structure learning in turn. Before continuing we would like to add a note of caution. We refer to our stimuli with very general terms, i.e., novel words and novel syntactic structure. This is in order to highlight a crucial difference between the conditions, namely the mapping of a lexical concept on a novel word form vs. the mapping of a thematic relations onto a structural relation. Learning of other types of words (e.g., verbs, adjectives) or structures (e.g., agreement, relative clauses) might lead to a different pattern of results—the investigation of which is beyond the scope of the present research.

### Common network for initial stages of learning

The first learning block, which yielded the largest performance gain across both the learning of words as well as sentence structure led to intriguingly similar brain activations across conditions. This overlap speaks for the validity of the *learning generality hypothesis*. The network of areas that showed decreasing activations over time largely corresponds to the domain-general network that has been proposed by Chein and Schneider (2005) as reflecting practice related changes in mechanisms of cognitive control and working memory. In this framework it has been assumed that domain general processes such as working memory, selective attention and performance monitoring support initial stages of learning until consistent associations are formed. At later stages of learning these areas were shown to fade out (Chein and Schneider, 2005). The domain general network that we found, comprised ventrolateral, dorsolateral and medial prefrontal areas, basal ganglia, temporal, parietal and cerebellar areas, the specific functions of which we will sketch in the following.

Both ventrolateral (VLPFC: BA44, BA45, BA47) and dorsolateral prefrontal cortex (DLPFC: BA9, BA46) have been found to be crucial for working memory processing. The DLPFC is thought to specifically subserve executive aspects of working memory such as manipulating and reordering of content in contrast to rehearsal processes, which are thought to be controlled by VLPFC cortex (Paulesu et al., 1993; Owen et al., 1996; D'Esposito et al., 1999). Linguistic candidate mechanisms that have been localized in VLPFC are strategic phonological processing (Poldrack et al., 1999b; Wagner et al., 2000) semantic selection (Thompson-Schill, 2003; Schnur et al., 2009) or syntactic processing (Caplan, 2001; Fiebach et al., 2001). Further, there is a proposal to view the entire VLPFC as a unification space for morphological, semantic and syntactic information under the influence of memory and control (Hagoort, 2005). Since executive functions and rehearsal in verbal working memory are indispensable for both the acquisition of new words and syntactic relations, we suggest these functions to be likely candidates for the present activations found for both learning conditions. Although the results of previous experiments suggest the dynamics in inferior prefrontal areas are different with respect to learning-related changes during the learning of syntactic rules vs. words, the present results point to changes in the same direction under similar learning conditions. A potential explanation for this might be related to the learning cues given in the previous AGL studies and in the present study. While all previous AGL studies provided feedback that allowed gradual extraction of syntactic rules from correct examples (Musso et al., 2003; Opitz and Friederici, 2003, 2004), the present study allowed much faster learning of the novel sentence structure due to the one-to-one mapping of the visually presented scene and the presented sentence. This means that both the meaning of the novel word and the interpretation of the syntactic structure could be inferred instantly, mapped onto the sentence and memorized. This overlap in learning principles may have been the cause for our finding that both learning of novel words and novel sentence structure was associated with prefrontal activation that decreased with increasing skills.

The current learning task also activated dorsolateral aspects of the premotor cortex (BA6). While this region has been classically related to preparatory motor functions (Wise, 1985), it became clear in the last decades that this region also contributes to linguistic functions in some way. Specifically, it has been shown to support comprehension of action-related language, possibly by a kind of mental simulation of the linguistic meaning of the utterance in an effector-specific manner (Hauk et al., 2004; Aziz-Zadeh et al., 2006; Willems et al., 2010, 2011). As the learning related activation that we found was located in dorsolateral parts of the premotor cortex, which have been shown to be related to the comprehension of manual action words (Willems et al., 2010, 2011) we suggest that participants used their premotor system to understand the depicted hand/arm action which assisted the extraction of the novel linguistic information.

Another part of the frontal cortex that was involved during learning was the pre-SMA extending to cingulate gyrus. In humans pre-SMA has been shown to be involved in many non-linguistic sequencing tasks such as action observation and selection, working memory or visual sequence processing and learning (Decety et al., 1997; Kennerley et al., 2004; Bahlmann et al., 2009; Schulze et al., 2009). However, pre-SMA also seems to play an important role during language processing both, during comprehension and specifically during production (e.g., Crosson et al., 2003; Rüschemeyer et al., 2006). In the light of these findings, the involvement of pre-SMA in our task reflects probably its contribution to the learning of the sequential aspects of the novel stimuli, that is syllabic/phonemic structure of novel words as well as word order.

In the vicinity of the frontal cortex activations, we also found learning related changes in the bilateral anterior insulae. Insular activation has been found across many sensory domains and cognitive tasks. In auditory experiments the insular cortex has been implicated in lower and higher level cognitive processes ranging from novelty detection, to verbal memory processing and phonological processing of words (see, for review, Bamiou et al., 2003). With respect to language processing the left anterior insula has been suggested to play an important role in articulatory planning, specifically during the production of novel or infrequent speech sounds (Dronkers, 1996; Carreiras et al., 2006). Across domains, the anterior insula plays a critical role in attention, working memory and higher functions of cognitive control (Dosenbach et al., 2007; Nelson et al., 2010) which might even be the function that is shared in both of our learning conditions.

Subcortically, we found learning-related decrease of activation in the pallidum bilaterally, which is part of the basal ganglia system. Basal ganglia activation was only reported in some previous word-learning studies (Mestres-Missé et al., 2008, 2009), but not in the AGL studies testing the acquisition of linguistic grammars (Tettamanti et al., 2002; Musso et al., 2003; Opitz and Friederici, 2003, 2004; Newman-Norlund et al., 2006). However, there is ample evidence from non-linguistic learning studies, that the basal ganglia play a prominent role during skill acquisition (e.g., Poldrack et al., 1999a, 2001; Seger and Cincotta, 2005; Cincotta and Seger, 2007; Ischebeck et al., 2007) as well as during native language processing (Mummery et al., 1998; Pickett et al., 1998; Moro et al., 2001; Kotz et al., 2003) and specifically, second language processing (Klein et al., 1994; Rüschemeyer et al., 2005, 2006). These results suggest that the basal ganglia are involved during domain general learning as well as effortful language processing which both play a role during the task at hand.

Within the parietal lobe, we found learning-related decrease of activation in superior parts (BA7). The superior parietal lobe (SPL) is a part of the association cortex that has been found to be involved in a variety of tasks among which are attentional processing (Corbetta et al., 1995; Corbetta and Shulman, 2002) and also short-term (see, for review, Wager and Smith, 2003) and long-term memory processing (see, for review, Ciaramelli et al., 2008). As pointed out in a meta-analysis by Wager and Smith (2003) the SPL has primarily been found during working memory tasks when the task implied executive demands such as ordering or manipulating the memory contents. This interpretation fits well with our task and data. As activation of the SPL was present from the first learning block onwards, it is most likely related to executive functions during working memory processing as long-term representations were not established yet in beginning states of learning.

Within the temporal lobe, we found learning related decrease in activation in the left superior temporal sulcus (BA 22). This area frequently appeared in studies of language comprehension at the phoneme level (DeWitt and Rauschecker, 2012), at the word level (Rissman et al., 2003; Okada and Hickok, 2006) and at the sentence level (Friederici et al., 2003, 2009; Pallier et al., 2011). Further, some of the studies on the learning of words also reported activation of temporal cortical areas (Mestres-Missé et al., 2008; Rauschecker et al., 2008; Davis et al., 2009; Paulesu et al., 2009), whereas studies on AGL and processing did not report activation in this area (Tettamanti et al., 2002; Musso et al., 2003; Opitz and Friederici, 2003, 2004; Friederici et al., 2006; Bahlmann et al., 2008). This suggests that the involvement of superior temporal areas in the present study is related to lexical-semantic aspects of the learning task or integration of syntactic and semantic information during sentence comprehension (Friederici et al., 2009).

The left lingual gyrus is a visual processing area that has also been related to higher cognitive functions such as visuospatial working memory and declarative memory retrieval (Ragland et al., 2002; Burianova et al., 2010). With respect to language processing it has been shown to be involved in reading (Mechelli et al., 2000) as well as in naming tasks using visually presented objects (Hocking et al., 2010; Liu et al., 2010). We suggest that the involvement of the lingual gyrus during our learning task is due to the requirement to use the visuo-spatial information in the picture in order to extract the meaning of novel words and the novel sentence structure.

Decreasing activation during the course of learning was also found in the cerebellum. Aside from its important motor functions, the cerebellum has been found to be involved in a variety of non-motor cognitive tasks (cf. Desmond and Fiez, 1998; Strick et al., 2009, for review). Specifically relevant for the present study, cerebellar activation has consistently been reported for a variety of verbal working memory tasks (cf. Wager and Smith, 2003; Wager and Smith, for review) as well as phonological word learning tasks (Rauschecker et al., 2008; Paulesu et al., 2009). Both of our learning conditions drew heavily upon verbal working memory resources and thus it is no surprise that the cerebellum is part of the observed network.

### Different temporal dynamics across learning conditions

In the present experiment, there was almost no difference between the learning of words and sentence structure during initial stages of learning which we took as evidence for the *learning generality hypothesis*. However, at later stages of learning, many areas, including inferior, middle and medial frontal and parietal cortices, showed a different course of activation changes over time across the two learning conditions. Learning of novel words showed larger activations compared to sentence structure learning in second, third and fourth learning block in fronto-parietal areas. Likewise, the novel sentence structure condition yielded increased activations in the last learning block compared to the novel word condition—mainly in temporal, medial frontal and posterior cingulate cortex, and the precuneus. We take this finding to suggest that after initial stages of extracting the novel words' and sentence structures' meaning different cognitive strategies are used to process and further consolidate what has been learned. This speaks for the validity of the *learning specificity hypothesis* for more advanced stages of language learning.

The main cognitive demand in the novel word condition is the successful encoding, storage and retrieval of a single novel word form and its meaning. As there were more single items to keep in memory in the novel word condition compared to the novel sentence structure condition, it is plausible that attentional and memory processes were challenged more and over a longer time span. In fact, this corresponds to common conceptualizations of word vs. rule learning that are found in the literature. Rule learning has sometimes been characterized as an abstraction process that operates very fast (Marcus et al., 1999; Peña et al., 2002) while word learning has been conceptualized, at least in part, as a probabilistic, associative learning process (Saffran et al., 1996; Breitenstein et al., 2005; Regier, 2005; Estes et al., 2007). The observation of a prolonged activation of fronto-parietal areas for the novel word condition suggests that similar brain systems contribute to the word and sentence structure learning, as discussed in detail in the preceding paragraph, but that linguistic representations emerge in a distinct manner, namely gradually for novel words and rather instantly for novel sentence structures. As the present study showed the effects in a naturalistic but somehow confounded learning setting where the participants are exposed to many more novel words than novel structures, future studies should aim to test if and how the activations are modified when the numbers of words an syntactic structures are kept constant.

For learning the novel sentence structure, activation in a different network emerged after initial stages of acquisition. The areas that we observed to be increased for sentence structure learning during the last learning block were located in medial prefrontal, posterior cingulate and bilateral temporal cortex as in the precuneus. Specifically the medial cortical areas are not classically reported for working memory and language tasks. However, strikingly similar patterns were found, when language had to be processed beyond the single sentence level, as for example during dialogue or narrative texts, in which pragmatic and contextual information plays a prominent role (Ferstl and von Cramon, 2001, 2002; Xu et al., 2005; Hasson et al., 2007; Yarkoni et al., 2008; Whitney et al., 2009). In our task, linguistic input (sentences) has to be integrated with non-linguistic contextual information (pictures), from which a situation model can be built, and thus, the language processing system might be taxed in a similar way as during text comprehension, where sentences have to be integrated with previously presented sentences. Compared to the sentences containing only a novel word, the situation model that the participants have to take into account during learning of a novel sentence structure is much more complex. The whole triadic interaction presented in the picture has to be represented in order to map the sentence correctly onto the scene. For the novel word condition, a narrow focus on the inanimate object suffices. We thus suggest that the increased activations for the novel sentence structure condition in the last learning block might be due to participants' successful mapping of the situation model built from the picture with the learned passive sentence. With respect to the functions of the specific sub-regions that appeared in this contrast it has been suggested that the medial prefrontal cortex supports integration of information during higher-order language processing, such as inference processes and coherence building (Ferstl and von Cramon, 2002; Xu et al., 2005; Hasson et al., 2007) in concert with the posterior cingulate gyrus and the precuneus which support visual imagery and memory processes that form the basis for higher order cognition (Binder et al., 2009; Mar, 2011). Linguistic functions assigned to the anterior temporal lobe are combinatorial processes in the semantic as well as in the syntactic domain (Hickok and Poeppel, 2007; Ferstl et al., 2008). During story comprehension the activations were sometimes found to be bilateral or even right focused (Mazoyer et al., 1993; Robertson et al., 2000; Ferstl et al., 2008). The right hemispheric activation that we observed in the present study might be related to the non-linguistic aspects of the combinatorial task, i.e., forming a situation model from visual input. Taken together, the novel sentence structure condition seems to specifically recruit brain areas that have been implicated in higher level linguistic and non-linguistic integration processes. This might be due to the higher complexity of the decoding and mapping of the picture and the sentence content. Notably, this specificity only emerged at a high stage of proficiency.

With the finding of common areas for earlier stages of learning and differences at later stages of learning, the current study suggests a common neural substrate that assists initial stages of learning across the linguistic domains of the acquisition of words and sentence structure by providing working memory and control functions. Over time, content-specific reallocations of brain resources occurred which shows the emerging neural differentiation of semantically vs. syntactically guided mapping processes.

## Funding

This work was supported by a fellowship grant from the Japanese Society for the Promotion of Sciences (PE07544) to Jutta L. Mueller.

### Conflict of interest statement

The authors declare that the research was conducted in the absence of any commercial or financial relationships that could be construed as a potential conflict of interest.
